# “I Felt Like I Was Cut in Two”: Postcesarean Bodies and Complementary and Alternative Medicine in Switzerland

**DOI:** 10.1007/s11013-024-09856-6

**Published:** 2024-05-06

**Authors:** Caroline Chautems

**Affiliations:** Center for Gender Studies, Institute of Social Sciences, University of Lausanne, Lausanne, Switzerland

**Keywords:** Switzerland, Cesarean, Postpartum, Cesareaned bodies, Complementary and alternative medicine

## Abstract

In neoliberal cultural contexts, where the ideal prevails that female bodies should be unchanged by reproductive processes, women often feel uncomfortable with their postpartum bodies. Cesareaned women suffer from additional discomfort during the postpartum period, and cesarean births are associated with less satisfying childbirth experiences, fostering feelings of failure among women who had planned a vaginal delivery. In Switzerland, one in three deliveries is a cesarean. Despite the frequency of this surgery, women complain that their biomedical follow-up provides minimal postpartum support. Complementary and alternative medicine (CAM) therapists address these issues by providing somatic and emotional postcesarean care. CAM is heavily gendered in that practitioners and users are overwhelmingly women and in that most CAM approaches rely on the essentialization of bodies. Based on interviews with cesareaned women and with CAM therapists specialized in postcesarean recovery, I explore women’s postpartum experiences and how they reclaim their postcesarean bodies.

## Introduction

In 2021, an ad from the US company Frida Mom, which specializes in postpartum care, was rejected for the Oscar ceremony for being “too graphic” (Shammas, 2020). In reaction to this decision, sociologist Weizman (2021) and friends^1^ initiated a movement on social media with the hashtag “#MonPostPartum,” aiming to break the taboos related to the embodied postpartum experience (Herzog, 2020). Using this hashtag, women are opening up about their experiences and posting photos of their postpartum bodies. By sharing pictures of painful, marked, leaking bodies, sometimes wearing disposable underwear, these advocates are making visible bodies that are usually invisiblized. In high-resource societies, where women are expected to “get their bodies back” shortly after giving birth, postpartum bodies are associated with a liminal, temporary, and subversive state. Bodily fluids in particular are stigmatized and thought of as dirty or contaminating (Bramwell, 2001). These secretions conjure representations of an out-of-control female body that have been inherited from the Enlightenment era (Kukla, 2005; Martin, 1987).

In Switzerland, as in most high-resource countries, pregnant bodies are regulated through a range of biomedical exams and via lifestyle and behavior recommendations aimed at controlling risks and optimizing fetuses’ health (Ballif, 2020; Manaï et al., 2010). Pregnant women are expected to gain “appropriate” weight and to favor exercise and a “healthy” diet (Jarty and Fournier, 2019; Kenney and Müller, 2017). Epigenetics research heightens mothers’ accountability by focusing on the impacts of the periconceptional socioeconomic environment on children’s long-term health (Kenney and Müller, 2017; Richardson, 2015). After childbirth, women are still expected to make their bodies constantly available to their babies, such as by breastfeeding, to ensure their secure attachment and optimal psycho-emotional development (Chautems, 2022; Faircloth, 2013; Kukla, 2005).

During the postpartum period, women receive confusing messages. They are expected to provide for their child, and healthcare professionals particularly insist on breastfeeding, identified by health authorities as the most beneficial infant-feeding mode (WHO, 2021a). However, women are simultaneously expected to regain control over their bodies, which is in line with a cultural ideology of female bodies remaining fit, smooth, and practically unaltered by the reproductive process. This idea of “getting the body back” after birth (Fox and Neiterman, 2015; Neiterman and Fox, 2017) also refers to injunctions to swiftly resume sexual intercourse, preferably penetrative penis/vagina sex following a cis heteronormative order. Accordingly, a lack of sex drive during the postpartum period is problematized as a biomedical issue (Hirt, 2009).

Although most women experience some degree of discomfort with their postpartum bodies (Fox and Neiterman, 2015; Neiterman and Fox, 2017), women who have given birth by cesarean are facing specific difficulties. In comparison with women who have given birth vaginally, they experience more pain, which some report as a serious issue that interferes with their daily activities months after the surgery (Declercq et al., 2008). Severe sexual dysfunction is also more frequent after a cesarean birth than after an uncomplicated vaginal delivery (Baud et al., 2020). Moreover, women delivering by cesarean are less satisfied with their first moments with their newborn and their overall childbirth experience (Bossano et al., 2017) than those delivering vaginally (Guittier et al., 2014). Women who had an emergency cesarean express the strongest dissatisfaction (Burcher et al., 2016; Guittier et al., 2014) and are at greater risk of posttraumatic stress disorder (Deforges et al., 2020). Paradoxically, regarding the degree of biomedicalization in high-resource countries, a vaginal birth, considered the “normal” delivery mode, is socially valorized as an “accomplishment” (Malacrida and Boulton, 2012; Miller, 2007). Additionally, the “natural childbirth” discourse is imposed as a dominant narrative to prepare pregnant women for vaginal delivery, including in prenatal classes (Malacrida and Boulton, 2014; Miller, 2007). Therefore, most women are planning a vaginal, “natural” childbirth, and undergoing a cesarean is associated with feelings of failure, disappointment, and/or grief (Chautems, 2022; Fenwick et al., 2009; Fox and Neiterman, 2015). In contrast, from a Global South perspective, accessing a cesarean often appears as a positive indicator of a privileged social status (Béhague, 2002; Cindoglu and Sayan-Cengiz, 2010; Klimpel and Witson, 2016; Maffi, 2012). Additionally, women’s representations of delivery modes vary and are shaped by local gender regimes and moral definitions of femininity and “good” motherhood (Maffi, 2012).

In Switzerland, almost one in three children are born by cesarean (32.7% in 2021, according Obsan 2023), placing the Swiss cesarean section rate among the highest national rates in Europe (WHO 2021b). This high rate reflects a risk-oriented and technocratic obstetric culture (Davis-Floyd, 2018, 2022; Maffi, 2012) that is characteristic of most high-resource countries (Davis-Floyd, 2018). Furthermore, the evidence-based medicine paradigm dominates healthcare systems and medical facilities, leading to a standardization of care based on hospital protocols, which increases biomedical interventions, including cesarean births (Downe and McCourt, 2008; Wendland, 2007). The rate varies significantly across Swiss cantons—from under 20% in Jura to 40% in Glaris—and healthcare coverage—45.6% for the minority of women with private insurance, who most often give birth in private hospitals, versus 30.7% for those with standard health insurance (OFS, 2019). A 2013 report from the Federal Office of Public Health indicated that the Swiss cesarean rate (then 32.6%) results from a wide range of factors, biomedical, sociodemographic, socioeconomic, and legal, and that the weight of each factor was impossible to assess due to a lack of reliable data. (Hanselmann and Von Greyerz, 2013, p. 28).

In line with a dominant “child-centered” parenting culture (Chautems, 2022; Faircloth, 2013; Hays, 1996), the cesarean section is primarily perceived as a mode of delivery and not as a major surgery that necessitates adequate care (Wendland, 2007). Newborns’ health and well-being appear as caregivers’ absolute priorities, and maternal recovery is often glossed over (Davis-Floyd, 2022; Lupton, 2012; Maffi, 2012). Wendland (2007) argued that birth technologies, especially the cesarean, led to the “vanishing” of mothers. Although biomedical technologies have been constructed in medical discourses since the twentieth century as safer for fetuses, women’s subjective experiences of birth and postpartum recovery are absent from the decision-making process of performing a cesarean in evidence-based obstetrics. In addition, medical studies have focused on the moment of birth and the following days and have not considered long-term complications and discomfort for women (ibid. 2007). As a result, standard biomedical follow-ups often fail to address the specific concerns women face after a cesarean delivery. To address these issues, complementary and alternative medicine (CAM) therapies have developed in the Swiss perinatal landscape: osteopaths, acupuncturists, and other therapists specializing in providing somatic and emotional care for women who have experienced a—sometimes traumatic—cesarean delivery.

Based on in-depth interviews with 26 cisgender women who gave birth by cesarean and 17 therapists specialized in postpartum and postsurgery care in French-speaking Switzerland, in this article, I explore women’s feelings, experiences, and care practices regarding their postcesarean bodies. In the absence of guidelines from biomedical health professionals, how do they handle their symptoms—somatic and emotional—to become one with their bodies again? How do CAM therapists help them in this process, and what types of care are they offering? Providing socioanthropological insights into women’s postcesarean healing trajectories, I aim to make visible the subjectivities of women who have “vanished” from evidence-based obstetrics (Wendland, 2007). This article will also contribute to the socioanthropological literature on the postpartum period, which has long been an understudied area in the research on reproduction (Neiterman and Fox, 2017).

In this article, which, again, is based on interviews with heterosexual cisgender women, I will use the term “women” as an analytical tool and a political category that is entrenched in the Swiss gender regime and in heteronormative culture. However, it is important to acknowledge that people who do not identify as women also experience cesarean births and recoveries. Furthermore, I will discuss cis heteronormativity in a way that calls for medical research and clinical practices that are inclusive of a spectrum of gender and sexual orientations.

## CAM Therapies, Gender, and Embodiment

In Switzerland, as in other high-income countries, CAM usage has increased over the last three decades (Eisenberg et al., 1998; Klein et al., 2015), with 28.9% of users aged 15 or older in 2017 (Federal Statistical Office, 2019). This prevalence is higher (37.6%) in French-speaking Switzerland, where I conducted this study (ibid. 2019b). Covered by supplemental health insurances only, CAM remains a privilege of wealthy people. In Switzerland, health insurances are products of private companies, and customers are only reimbursed for healthcare costs once they reach the deductible, an amount that varies greatly depending on the coverage options. Maternity care is an exception, as all biomedical care is fully covered without deductibles from 13 weeks of pregnancy until 8 weeks after delivery. Supplemental health insurances do not include a deductible, and clients are usually reimbursed 80% of the costs. In this system, CAM therapies may turn out to be cheaper than biomedical care, which encourages CAM users who already have a supplemental insurance to intensify their CAM usage.

CAM’s growing success has been associated with patients’ dissatisfaction with biomedicine and the underlying power relationships between physicians and patients as well as with a valorization of patients’ subjective and embodied experiences (Sointu, 2011). Based on a definition of health as a state of well-being, defined as “holistic” (Sointu, 2011), in CAM therapies, the body becomes a “reflexive project” and identity is built on individuals’ lifestyles and everyday practices (Giddens, 1991). These processes align with the emergence of a “therapeutic culture,” “a culture focused on exploring the stories, needs, and dysfunctions of individuals,” which Klassen (2001, p. 67) linked to the decline of institutionalized religions and the rise of alternative spiritualities. Following a neoliberal understanding of identity, the self appears as a promise of fulfillment if adequately guided by “experts of the soul” (Rose, 1998, p. 17), such as CAM therapists.

Similar to other industrialized countries (Frass et al., 2012; Keshet and Simchai, 2014), in Switzerland, women are overrepresented in CAM, both as consumers (Federal Statistical Office, 2019; Klein et al., 2015) and as therapists (Dubois et al., 2019). CAM is partly intertwined with “holistic spiritualities” (Sointu and Woodhead, 2008), a range of body-centered practices that has grown since the 1980s, and endorses the unity of body, mind, and spirit, with the aim of improving individuals’ well-being. Holistic spiritualities overlap with CAM, as CAM therapies often include a spiritual component and are influenced to various degrees by the New Age movement, especially those relying on the notion of “energy,” such as acupuncture, massage, and energy therapies. Women often easily engage with holistic spiritualities and with CAM, either as users or as practitioners, because these approaches value the traditional gendered roles of healthcare providers (Keshet and Kimchai, 2014; Sointu, 2011; Sointu and Woodhead, 2008).

Moreover, CAM is associated with gendered stereotypes, as it is usually considered a “gentle,” more feminine, and comprehensive health approach, compared with the “rigidity” of biomedicine’s technocratic approach (Davis-Floyd, 2018, 2022; Keshet and Kimchai, 2014; Sointu, 2011). CAM therapies offer individualized care that fosters feelings of involvement and partnership, in opposition to biomedical hierarchies of patient and physician (Keshet and Kimchai, 2014). Therefore, CAM also emphasizes individuals’ responsibility in achieving health, relying on autonomist values that are characteristics of neoliberal regimes (Sointu 2013). However, this stress on individual responsibility regarding health management is also predominant in biomedicine; once informed about health risk prevention and good practices, individuals are expected to practice self-discipline (Memmi, 2004; Rose, 2006).

Sointu (2011) argued that through this focus on women’s individual fulfillment and agency, CAM forms “a feminised setting that also conflicts with traditional discourses of other-directed femininity” (357). From this perspective, CAM would contribute to a “detraditionalization” of gender roles, as it subverts the “being for others” injunction that is often addressed to women (ibid. 2011). On the contrary, Brenton and Elliott (2014) asserted that the ways women and men use CAM therapies reinforce traditional gendered roles. Furthermore, Longman (2020) demonstrated that the “entrepreneurism of the feminine” has been spreading over the last decade, fostering an essentialization and sacralization of female embodied processes and experiences. Keshet and Kimchai (2014) named these contrasting interpretations of women’s prominence in CAM a “gender puzzle,” “largely based on the life experiences of privileged women and on issues of self-actualization, empowerment, identity formation, and autonomy, while adopting the rules of the global neoliberal economy” (85). From an intersectional perspective, CAM reproduces both race and class privileges, as its users remain predominantly white and wealthy people (Longman, 2020). In addition, CAM often includes care practices that stem from the traditional medicines of Global South cultures, such as India and China, and that are adjusted to fit into the healthcare markets of Global North cultures. Nevertheless, women in low-income countries often rely on traditional medicine as an affordable alternative to biomedicine (Broom et al., 2009; Cameron, 2010).

The socioanthropological literature on CAM includes explorations of women’s CAM usage but not examinations of how a specific market developed for the perinatal period. In addition, CAM gender dimensions have been debated, but rarely from the users’ embodied perspective. Focusing on women’s postcesarean healing experiences, I aim to address these gaps.

## Methods and Participants

This article is based on in-depth interviews with 26 Swiss white cisgender women who gave birth by cesarean and used CAM therapies during the postpartum period and with 17 therapists of various CAM modalities in French-speaking Switzerland. I first contacted CAM practitioners, and they referred me to some of their clients and colleagues. The interviews took place from April 2021 to December 2022 and lasted on average 1.5 h. Depending on the participants’ preferences, I conducted 25 interviews by video conference and 18 in person. I secured informed consent from my interlocutors, and the Swiss cantonal ethics committee on research involving humans approved this study. The study presented in this article is part of a larger study on parents’ experiences of cesarean births in Switzerland. Consequently, the interviews with mothers included their overall experience, from pregnancy to the postpartum period. In my interviews with CAM therapists, I explored the trajectory that led them to provide care for cesareaned women, as well as an in-depth description of their care and their experiences of and observations about the issues their clients faced. The interviews were recorded then transcribed and prepared for a thematic analysis (Beaud and Weber, 2010). All data were coded using a spreadsheet to enable the identification of recurrent themes. Based on my early interviews, I defined emergent themes (e.g., relationship with the scar) and established a first coding framework. I then refined the categories and added new ones (e.g., postpartum sexuality) as I progressed with my interviews and as my interpretations of the data evolved. Following an inductive approach, I built my analysis progressively and iteratively by circling between interviews, my analysis spreadsheet, and socioanthropological literature. This means that the data collection overlapped with data analysis. This circular approach enriched each stage of the study with input from the others (e.g., injecting insights from first analyses while conducting later interviews). All my interlocutors spoke French, and I translated their words into English. All names are pseudonyms, and all identifying details have been modified.

The mothers who participated in the study had given birth 6 months to 24 years before the interview: 5 had a cesarean less than 1 year before the interview, 16 had a cesarean between 1 and 5 years before, and for 5 participants, it had been more than 5 years. This diversity allowed me to include a temporal dimension in women’s recovery trajectories and to provide a long-term perspective on some persistent postcesarean issues. Eleven women had multiple cesareans. Altogether, my interlocutors underwent 38 cesareans, of which 30 were emergencies and 8 were planned. In general, emergency cesareans were often traumatizing experiences, from both somatic and emotional perspectives. Since I met them through their CAM therapist, all mothers used CAM therapies during the postpartum period to address somatic and emotional issues caused by the surgery. For this study, I only met with women who were dissatisfied with their recovery, which is of course not the case for every cesareaned woman. They often mixed methods and did not limit themselves to one care approach. The majority of them were already regularly consulting CAM therapists before becoming pregnant. However, the frequency of consultations often intensified during the perinatal period. In general, my interlocutors fell within Schmitz’s (2006) definition of “medical pluralism,” which is characteristic of high-income countries’ healthcare markets: idividuals use CAM therapies together with biomedicine and switch from one approach to another.

The participants were between 27 and 70 years old. All of them were part of a heterosexual couple with their child’s father. On three occasions, I conducted the interview with the couple; in all other instances, only the mother participated. Interestingly, in the couple interviews, the fathers were loquacious about how they experienced the cesarean birth, but they mostly remained silent about postcesarean recovery and their partner’s feelings about their postcesarean bodies, so their voices do not appear in this article.

Most women had a middle-class background and a higher education degree. This profile is consistent with average CAM use in Switzerland (Federal Statistical Office, 2019) and other high-income countries (Bishop and Lewith, 2010; Upchurch and Chyu, 2005). CAM users identify as predominantly white (Bishop and Lewith, 2010; Keshet and Simchai, 2014; Upchurch and Chyu, 2005). In my study, all participants (users and practitioners) were white women.

All the therapists I interviewed were Swiss white cisgender women between 31 and 59 years old. Most of them worked part time, and almost all of them were living with a working partner. Keshet and Simchai (2014) argued that this profile, which is common in CAM, proceeds from part-time training to part-time work, thereby explaining the prevalence of women in CAM. These modalities are compatible with gendered domestic organization and with women’s burden of being the primary care provider in the household, as is still the case in Switzerland (Bornatici et al., 2021).

CAM therapies are often marketed as empowering women by promoting female entrepreneurship, whereas therapists generally rely on their (male) partner’s income (Keshet and Simchai, 2014). Interestingly, several therapist participants had a contrasting profile. They worked full time and had strong collaborations with biomedicine through hospitals or private obstetricians. This was particularly the case for osteopaths, whose profession has been taught in biomedical schools since 2014. Switzerland is the only European country that has deprivatized and uniformized osteopathy training.^2^ Some therapies the practitioners I met offered were partially reimbursed by supplemental health insurances, whereas others were not covered by any health insurance, including the Grinberg method (which is meant to enhance body awareness), kinesiology (a mind–body balancing technique based on “muscle testing”), and energy therapy. When I asked therapists about this issue, some argued that a single session is generally sufficient, which minimizes the cost of their services. In line with a neoliberal ideology, others felt that cost is part of the healing process’s success. One of the Grinberg therapists I interviewed claimed, “Personal motivation is very important to the success of the work. The fact that they pay from their own pocket is important in the therapeutic outcome.”

The therapist interlocutors were from a large variety of approaches and trainings: four osteopaths, four practitioners of the Grinberg method or “body awareness,” three acupuncturists, and six therapists from other approaches (energy therapy, kinesiology, massage therapy, naturopathy, or reflexology). Some of these CAM approaches rely on elaborated theoretical backgrounds and several years of full-time training, whereas others require only short part-time trainings and rely on the notion of “the gift” or individual sensitivity. Beyond these divergences, which are outside the scope of this article, I found similarities in the ways the CAM interlocutors identified women’s postcesarean needs and specific difficulties. Some practitioners specialized in postcesarean care, often following a personal experience that led them to design the type of care that they missed out on when they were recovering from a cesarean. Other practitioners specialized in perinatal care and followed women postcesarean, among other types of clients. Overall, my interlocutors were eager to denounce the lack of support from biomedical professionals in addressing the specificities of cesarean recovery. As one osteopath specialized in intrapelvic osteopathy stated, “It’s probably because no one talks about it that I wanted to take an interest in it. Postpartum is the poor relation of medicine.”

## Healing the Scar, Becoming One Again After “Being Cut in Half”

Based on my interviews with women and with CAM therapists, I can say that, in addition to increased pain in comparison to a vaginal delivery, postcesarean consequences include long-term issues linked to the scar and its healing. Scar tissue adherence is very common and may lead to a loss of bladder and uterus mobility (Poole, 2013). As a result, women complain of bladder discomfort, pain during penetrative intercourse, digestive troubles, and backache. In the scar area, they also experience insensitive or itchy skin for months after the surgery or even permanently. Interlocutors who had undergone a cesarean over 15 years before our interview still experienced discomfort with their scar.

My interlocutors complained that healthcare professionals often minimized the persistent pain that they reported, as it was not threatening their or their babies’ health. For example, Carole suffered from discomfort for months after her emergency cesarean birth. At her 6-week postpartum consultation^3^ with her gynecologist, she expressed that she was hurting: “I had a lot of pain, but she said that it was normal. I was in pain for more than 1 year. It didn’t wake me up at night, but it was showing up at the end of the day. The scar hurt. I had seen osteopaths who had worked on it, it helped, but the pain returned; it was not going away.” One of the osteopaths she consulted suggested that she contact a practitioner of the Grinberg method who specialized in postcesarean recovery. The pain eventually faded after only two sessions with this practitioner.

The therapists I met regretted that biomedical healthcare providers do not adequately support women regarding care for their scars. CAM therapists believe that massages are essential for increasing elasticity and comfort and reconciling women with their postcesarean bodies. The women interlocutors who had planned a vaginal birth were often disgusted by or resentful of their scar, seeing it as a manifestation of their failure to give birth naturally. Consequently, many women are unable to touch their scar or even look at it. Emma said, “Initially, I didn’t like looking at my scar. . . . It took me a long time to stop resenting it. Later, watching it would not disgust me anymore, but it took me a while to look at my scar without strong emotions.” Aude, a Grinberg method practitioner, created a group workshop dedicated to postcesarean recovery. Based on her observations of women’s “disgust and alienation” regarding their scar, she purchased small rubber balls to teach women how to massage their scar:

It allows them to touch the scar without touching it directly. For many women, it is a way to reconnect with a part of their body that was injured, where there was an intrusion, and be able to say to themselves, “It is closed now, it is good.” Some people felt like it would open up if they made a wrong move. The idea behind massage is to strengthen that area because that strength gives you confidence.

In general, therapists highlighted the benefits of the “therapeutic touch,” especially in cases of chronic, medically unexplained pain, as experienced by some women after a cesarean. Lisa, an osteopath specialized in urogynecology and perineology, uses intravaginal techniques. Discussing the notion of “therapeutic touch,” she said,

As part of an osteopathic treatment, you have to consider both the effect of the treatment itself and the effects of the therapeutic touch, through which I validate the pain they feel, a pain not always understood—one hand in, one hand out—to restore flexibility. Just touching helps. Touching the places where the operation occurred that are painful, with precision, validates their distress.

In addition to somatic care, CAM therapists always conduct an in-depth debriefing of the cesarean birth. Therapists emphasized that women’s relationship to their scar provides a relevant gauge of their cesarean experience and of the status of their emotional recovery. An inflamed scar would then often be correlated with a traumatic delivery. Women interiorized these explanations. For example, Sophie had an emergency cesarean and was not properly anesthetized during the operation. She ended up with general anesthesia. Traumatized by her delivery, she “hated” her scar and was unable to touch it for weeks: “The scar looked puffy; I think it’s because I hadn’t accepted at all that I gave birth by cesarean.”

Therapists also observed that working on a woman’s scar often triggers strong emotions. These manifestations are perceived as beneficial and as necessary steps in the healing process. One energy therapist, Sylvia, explained the following:

You can feel tissue adherences when you touch the scar. This is often linked to something emotional, which remains stuck in the scar. When it’s an emergency, a cesarean birth is very difficult to accept. There is often a lot of anger, a lot of guilt, and a feeling of incompetence. When I touch these areas on the scar, people cry, and it’s a release. And you can see it. The scar changes. You can see the change in just one session. As soon as the emotional part is cleared, the scar can heal.

As the most extreme form of technocratic childbirth (Davis-Floyd, 2018, 2022), an unplanned cesarean birth often causes women to feel alienated from their bodies. It is associated with increased somatic and emotional difficulties in the postpartum period in comparison with a planned cesarean birth (Burcher et al., 2016; Deforges et al., 2020; Guittier et al., 2014). Many of the women I met shared feelings of powerlessness and dispossession resulting from their cesareans, often expressed by the idea that doctors “tore” their baby out of their stomach. CAM therapists validate these feelings. From their perspective, when the cesarean birth experience is more difficult, so is the recovery, which is also true at the somatic level.

Moreover, in a cultural context that values vaginal childbirth as an empowering experience, a cesarean birth could threaten a woman’s perception of her femininity (Malacrida and Boulton, 2012). An acupuncturist commented, “If childbirth didn’t happen as planned, it’s like a slap in the face. Working on her scar allows me to give the power back to a woman who has been slammed. It’s a matter of identity; they feel humiliated.” Some therapists accentuated that the incision was made in an intimate body part, linked to femininity and sexuality, which one massage therapist called “the sacred area of a woman’s body.” This location could complexify the healing process. An energy therapist emphasized that a cesarean birth deeply affects women, unsettling their femininity: “There is a loss of femininity, a loss of sexuality, and a loss of desire. It affects who they are. My treatment gives results: my patients find themselves as women again.” In this way, these therapists aligned with heteronormative biomedical discourses about a “healthy” and fulfilling sexuality (Gardey and Hasdeu, 2015), which also became a moral and social ideal. A massage therapist summarized her treatment as a way to “make peace with one’s scar, one’s cesarean, one’s feminine energy, and one’s sexuality.” In these discourses, femininity and sexuality are always intertwined, reproducing a heteronormative framework.

As highlighted by Malacrida and Boulton (2012), a vaginal birth implies that “a baby occupies a vaginal space normalized as solely appropriate to heteronormative sexual pleasure,” thereby posing a threat to the smooth resumption of penetrative sexual intercourse (751). In contrast, a cesarean birth would appear as protective of women’s sexual functions, regardless of medical research results showing that severe sexual dysfunctions are more frequent after a cesarean birth, in comparison with an uncomplicated vaginal delivery (Baud et al., 2020). As a part of a cis heteronormative framework, the main indicator of sexual health in biomedical studies remains linked to penetrative penis/vagina intercourse (Ollivier et al., 2020).

In addition, obstetricians often do not address sexuality during consultations in a way that meets women’s expectations; women would expect their obstetrician to bring it up spontaneously, thus opening up a space to discuss it at each consultation, whereas physicians fear that systematically raising the issue may be intrusive (Schweizer, 2017). As a result, postpartum sexuality remains mostly unaddressed in obstetricians’ follow-up consultations. Osteopath Lisa elaborated on this topic:

The problem is that, at the 6-week appointment, gynecologists should say, “Okay, you can resume intercourse, but if, after 3-4 times, it still doesn’t feel right, you have to come back and talk about it.” There is a gap about intercourse pain: They expect their gynecologist to talk about it. Patients don’t dare to address the topic, and neither do gynecologists. There is a huge gap because the subject is not discussed.

Beyond dyspareunia (painful sexual intercourse) and discomfort, during interviews, women often linked their postpartum sexuality to their birth experience and to their relationship to their postcesarean body. This echoes Ollivier et al.’s (2020) call for health professionals to address postpartum sexual health based on both physical criteria and emotional components.

Rebecca and her partner were planning to give birth in a birth center, with their midwife and with no medical interventions. They had carefully prepared the delivery that Rebecca had pictured as a moment of empowerment. She transferred to the hospital during labor and eventually had an emergency cesarean under general anesthesia for placental abruption. During our interview, she said that their sexuality as a couple had changed a lot since their child’s birth 2 years earlier: “I have this image that if you give birth vaginally, you are very powerful and very comfortable in your body. I had an operation and an incision, and we are both less at ease with my body. It’s a matter of relationship with the body.” In addition, some women remain uncomfortable with the appearance of the scar for years after the surgery. Anne had two cesarean births over 20 years prior to our interview. She described her stomach as an “apron” or loose flesh that folded over her cesarean scar. While undressing to show me her stomach and her scar, she elaborated as follows:

For me, the scar discomfort is linked to its effect on my stomach. My skin didn’t tighten. Above the scar, my belly is flat. I have no longer found myself in a desirable female body; my whole relationship to my body has changed. I lost 36 kg five years ago. And the apron remained. I was almost back to my wedding weight, but there was no way to tighten that belly. I never got my body back to where it was before because my belly took on that fold. Maybe I should have had an operation, but I didn’t want to have my belly cut again. I would have liked to be better accompanied—to do perineal rehabilitation . . . Acupuncture reduced the insensitivity. But I don’t feel my skin like I do on the other parts of my belly. Thankfully, I looked for other inputs in addition to what the doctors were telling me.

Undergoing a cesarean left a permanent mark on Anne’s body, which she linked to feeling “less desirable” as a woman, whereas, in her opinion, she would have been able to fully recover from a vaginal delivery. In a cultural context where women are expected to recover their prepregnant body after giving birth, cesarean scars put women in a position of failure; they may evolve and eventually fade, but they will always remain. It struck me during interviews that if emotional traumas seem to heal with time, in the longer term, some women still experience scar discomfort such as insensitivity, tensions, or insecurity regarding its appearance.

In relation to the location of the incision, the therapists I met often stressed the idea of a body “cut in half” after a cesarean: From an emotional or energetic perspective, the lower part of the body would be “dead.” According to these practitioners, working on the scar would “awaken” this lower half: Some women would, for example, “feel” their legs again. During the interviews, women also regularly referred to a feeling of being “cut” or disconnected from their lower body. Alexandra had an acupuncture treatment on her scar 20 years after her cesarean delivery because she had a persistent feeling of disconnection from her lower body:

When I started yoga, I felt that I was not touching the ground—that I had no legs—and this sensation of having the belly open with a huge energy that I was losing from there. With my yoga practice, it quickly became obvious that my legs were not touching the ground. It stopped at the pelvis.

In addition to yoga, Alexandra partook in women’s circles.^4^ One participant told her about Sabine, an acupuncturist specialized in obstetric scars, and Alexandra swiftly contacted her; she stated,

Regarding my feeling of being cut in two, she saw it on an energetic level. In terms of blood circulation, there was just a little bit of work to do. The big revelation was that there was a vertical scar underneath the superficial horizontal scar. I didn’t know that. That’s where it reacted the most with her needles. She wanted me to tell my story. There were needles all over the area of insensitivity, and there was a hole in the vertical scar. I felt the energy going down into the pelvis, going down into the legs. She massaged my legs, my feet, and I had the feeling that my legs were very long. When I left, I was hungry. And it felt very strange to walk. And then I saw the difference in my yoga practice. Very quickly I felt a whole other sensation in the pelvis. Something connected that was really alive and sometimes even orgasmic.

Sabine elaborated as follows:

A scar is like a stop in time. Women’s cesarean experience is inscribed in their scar. And when I work on it, it closes the loop while opening circulation physically. After that, the scar looks different. Some of them have sensations in their legs again, and some of them have sexual desires again. The technique is very simple, but it has a strong impact. It is both a psychotherapy and a bodywork. In general, one session is enough, except in cases of multiple surgeries. When I treat women, what comes out is the real trauma. Some women don’t understand why their hands were tied during the surgery or why the anesthesia failed. They grit their teeth, and that pain stays in the tissue. Either when the needles are in place or when I remove them, the woman lets go of all the pain. She entrusted physicians with her body because she had to, and she eventually recovers because she can finally speak.

Sabine emphasized women’s alienation as induced by a highly technocratic childbirth experience (Davis-Floyd, 2018, 2022). I also met women whose surgery was extremely painful due to anesthesia failure or who suffered other forms of obstetric violence. CAM therapists offered them a safe place to unburden and to reclaim their birth experiences.

## “The Baby’s Choice”: Recovering from Guilt

Although most CAM therapists focus their attention on maternal recovery, they also aim at enhancing new maternal identities while sustaining the mother–child relationship. As an embodied experience, vaginal delivery appears as a gauge of a woman’s suitability for motherhood (Malacrida and Boulton, 2012). From this perspective, the way in which delivery unfolds would be a harbinger of how a woman will achieve motherhood, and women often feel responsible should there be any deviation from their birth plan. Therefore, the therapists I met insisted on the importance of acknowledging women’s cesarean birth experiences. Lucile consulted a massage therapist 1 month after her cesarean:

I was very disappointed to have a cesarean. And she really comforted me by saying, “No, what you did is great. You had courage; you are a mother.” I needed to hear that; just because you had a cesarean doesn’t mean you’re not a mother.

In addition to feeling disempowered, women who had planned a vaginal delivery often feel guilty toward their child. These feelings stem from biomedical studies that associate cesarean births with increased health risks for children (Lagae et al., 2019; Sevelsted et al., 2014). For example, Mueller et al. (2015) argued that a cesarean birth negatively affects the infant’s microbiome, increasing risks of metabolic and immune diseases and affecting the infant’s long-term health. Although these findings are questioned (e.g., Chu et al., 2017), they are widely spread in media and social media, and they came up frequently during my interviews with parents. Some experts have also claimed that cesareans compromise children’s psychoemotional development. For example, Odent (2005), a leader of the natural birth movement, attributed “an alteration of the [child’s] capacity to love” to cesarean births, possibly leading to long-term social issues. Overall, these discourses tend to strengthen the guilt of parents whose baby was born by cesarean and who were initially anticipating a “natural childbirth.” Line felt that way and invested in breastfeeding as a way to make up for the perceived “harm” done to her daughter: “We know that when they are born by cesarean, they have a worse start in life; they did not have the microbiota of the mother, but at least breastfeeding worked well, and I was able to provide her with the benefits.”

Rachel had an emergency cesarean birth due to a liver dysfunction that was detected during a prenatal checkup 1.5 months before her due date. She elaborated as follows: “My first words to the midwife were that I felt like my baby had been ripped out of my belly. I kept blaming myself because I was sick, and he didn’t choose. I was focused on whether the baby was okay. I blamed myself a lot; I told myself it was my fault and that he wasn’t ready.” A few months after birth, she consulted a hypnotherapist who freed her from this guilt: “It helped me a lot. I only did one session; he found the blockage right away. It helped me accept that my baby understood that he had to get out.” Other mothers and therapists referred to this idea that babies have agency and contribute to the delivery decision process. From this perspective, which considers fetuses as conscious beings, some babies “choose” to be born by cesarean.

Some kinesiologists practice the so-called “Parole au bébé” (“baby’s speech”), an approach elaborated by Denis (2009), a Canadian perinatal kinesiologist. During a *Parole au bébé* session, the practitioner asks the baby closed-ended questions, and one parent acts as an intermediary. The practitioner exerts pressure on the parent’s arm and judges the baby’s answer based on the parent’s arm’s degree of resistance. Nathalie, a “Parole au bébé” therapist, elaborated on this topic:

Sometimes the cesarean is the baby’s choice. Just saying this to mothers gives meaning to their experience. Personally, it helped me a lot to understand that it was my son’s choice. Also, it is important for mothers to hear that it’s not necessarily a difficult experience for their baby. When I ask parents how the birth went, sometimes it was a beautiful birth, and yet the baby did not experience it well. And, quite the opposite, sometimes parents are worried after a difficult birth . . . And, in fact, when I debrief with the child, there is nothing wrong. For parents for whom it was important to give birth vaginally, there is already the ordeal of the cesarean itself and, on top of that, the guilt they feel.

According to Nathalie, babies would sometimes “choose” a cesarean birth because it seems safer or easier, and “understanding that” would remove responsibility from mothers. In the era of “intensive motherhood” (Hays, 1996), mothers are deemed accountable for every aspect of their child’s development and well-being, whereas fathers would only play a secondary role. Studies in developmental psychology that stress the damages of “maternal deprivation” (Bowlby, 1969) still occupy important positions in Swiss biomedical curricula. As a result, healthcare providers are particularly attentive to the adequacy of maternal behavior and almost exclusively attribute childcare responsibilities to mothers (Ballif, 2020; Vozari, 2015). Women’s feelings of responsibility and guilt are further strengthened by these factors, and from the therapists’ perspective, releasing these burdens would be an important part of recovery.

Moreover, this idea of the baby’s “choice” also relies on a shared belief among energy therapists and their clients that women cannot control the outcomes of their births. Isabelle told me that her openness to what she called “esotericism” eased her acceptance of her cesarean birth:

I realized that this was also my son’s story and it is important to differentiate it from mine. When you have an esoteric approach, it’s easier to not feel omnipotent and guilty. In the esoteric world [at birth], it seems that the incarnation process is difficult. The soul may want to go back and not incarnate, and now with these cesareans, we are actually able to go and get them. An esoteric approach helps to accept that we don’t control everything.

These spiritual beliefs, which Isabelle called “esoteric,” were shared by many of my interlocutors, such as acupuncturists, energy therapists, and their clients who accepted the notion of “energy” in their care approaches. These participants fit within Fuller’s (2001) definition of “spiritual but not religious.” They embrace a spiritual perspective, often identified in their discourses on “energy,” yet do not participate in an established religion.

All my interlocutors emphasized the connection between emotional and somatic recovery. Acupuncturist Sabine described her treatments as both “body work and psychotherapy.” Mind, body, and spirit are thought of as one unified energy field, which is characteristic of the CAM holistic approach (Davis-Floyd, 2018, 2022; Sointu, 2006). This connectedness extends to babies, as most therapists argue that healing mothers has positive impacts on children and on the mother–child bond. From this perspective, whereas CAM therapists claim to take a “woman-centered” approach, in actuality, it appears to also revolve around the child’s well-being, based on the assumption that “if mom is fine, baby is fine” (Vozari, 2015). This posture indicates therapists’ compliance with developmental psychology approaches, evaluating children’s well-being based on their mother’s serenity: mothers’ negative emotions must be “released,” or they will have a detrimental impact on their child. Therapists particularly insist on how their care benefits the mother–child relationship, which would be damaged after a traumatic birth experience. Some practitioners offer specific treatments intended for children born by cesarean, based on the belief that a cesarean birth could be detrimental to the child’s development. For example, Sylvia, an energy therapist, targeted children born by cesarean, arguing that,

In the birth process, we have autonomous movements [that are] designed for birth. And, in case of a cesarean birth, these movements are not activated, and it affects social skills. It can generate behavioral problems, such as hyperactivity. Children need to be reassured much more, as they lack confidence.

To “overcome this shortcoming” in the child’s experience, some practitioners such as Sylvia offer children the opportunity to experience childbirth again by reproducing the movements that they would have made during a vaginal birth. This therapeutic modality is sometimes called “rebirthing.”

## Conclusions

CAM therapists offer postcesarean care, thereby filling a gap in biomedical care. As CAM therapies are not covered by basic health insurances, their access remains limited to economically privileged women. Ideal neoliberal CAM clients are informed, self-determined, and responsible. Therefore, in the absence of a biomedical protocol, postcesarean recovery falls under individuals’ responsibility. Under the guise of “empowerment,” CAM contributes to a stratification of care access (Brenton and Elliott, 2014; Davis-Floyd, 2018, 2022; Keshet and Simchai, 2014). CAM reproduces both class and race privileges, and it remains largely inaccessible to marginalized populations.

During the research on which this article is based, I encountered a very diverse range of therapists, approaches, and trainings. Osteopaths’ practices stand out as pragmatic and empirical. None of them reasoned based on spiritual notions like “energy.” All therapists from other approaches openly referred to such concepts. In Switzerland, osteopaths’ status is unique among CAM therapists, as osteopathy is taught in public health schools and is covered by basic health insurance. However, beyond these divergences, all of my interlocutors’ discourses and services reflect a joint commitment: supporting the somatic and emotional recovery of cesareaned women, acknowledging their feelings, and helping them to reconnect with their postcesarean bodies.

Many CAM therapists focus on cesarean scars to facilitate both somatic and emotional recoveries because they conceive of the scar as the epicenter of women’s cesarean experiences. In both women’s and therapists’ discourses, CAM therapies appear as spaces of self-care, which is subversive in a context where mothers’ well-being is secondary in comparison to that of their child—the very source of lacking biomedical postpartum care.

CAM therapies also intend to reconcile women with their femininity and sexuality, thereby aligning them with the dominant biomedical narratives of a mandatory and fulfilling (hetero)sexuality (Gardey and Hasdeu, 2015). This conception of sexuality aligns with a “plastic sexuality” (Giddens, 1992), a concept that arose from the diffusion of contraceptive technologies and from the acknowledgement of women’s sexual desires and pleasures, whereas since the emergence of the medicalization of sexuality in the 1850s, medical discourses had considered that sexual drives were lower for women than for men (Gardey and Hasdeu, 2015). In the era of “plastic sexuality,” biomedical discourses also contribute to defining a norm of female pleasure, which in turn conditions what is expected of women in the context of cis heterosexuality (Gardey and Hasdeu, 2015). Furthermore, women’s sexual “functions” are thought of as incompatible with the maternal “function” that is thought to be superior to women’s sexual functions (Stearns, 1999). During the postpartum period, women are therefore experiencing tensions between a conception of their female bodies as “heteronormative sites of pleasure and sexuality” and as sites of boundless maternal nurturance and dedication (Malacrida and Boulton, 2012, p. 752); the “good maternal body” is an asexual body.

Based on these observations, I second Ollivier et al.’s (2020) pledge to healthcare professionals, including CAM practitioners, to “challenge dominant, medical, heteronormative or patriarchal sexual health and postpartum discourses,” and the restrictive measures of sexual well-being that they convey (i.e., penetrative sex), which are detrimental to postpartum women (people).

Consistent with Longman’s (2020) and Sointu and Woodhead’s (2008) studies, my research showed that CAM approaches were largely embedded within heteronormative norms of femininity and sexuality. While revalorizing female embodied processes and experiences, they are also reflecting an essentialist gendered framework and traditional views on gender roles (Brenton and Elliott, 2014).

Most CAM therapists argue that healing the mother has positive impacts on the child and therefore supports or “repairs” the mother–child bond. From this perspective, postcesarean care, while primarily devoted to women’s well-being, would also allow them to be better mothers, aligning with the ideology of “intensive motherhood” (Hays, 1996). Beyond somatic difficulties, cesareaned mothers suffer emotional and moral concerns; that is, they failed to achieve a vaginal delivery in a cultural context where the body is the means of good mothering (Faircloth, 2013).

As Neiterman and Fox (2017) observed about physical activity, for my interlocutors, engaging in CAM therapies, a form of body work, was also an endeavor to achieve a sense of control of their postcesarean bodies. As bodies are at the center of CAM approaches, for women whose bodies were invalidated at the time of birth, CAM therapies help them to revalidate their maternal bodies and to find their way back to their embodied selves.

The women I interviewed for this article had all experienced somatic and/or emotional difficulties postcesarean and resorted to CAM therapists to address them. It would be interesting to investigate the experiences of women who do not seek specific postcesarean care. As most of the participants had an emergency cesarean birth, more research is also needed to understand how the type of cesarean—planned versus emergency—influences the recovery process.

## Footnotes

1 A. Saura, M. Koresh and M. Sacré.

2 Since 2020, a new law on osteopathy has come into force, meant to standardize training at the federal level (LPSan 811.21).

3 This consultation ends the obstetrician’s perinatal follow-up. It is mainly intended to review the mother’s health condition, discuss perineum rehabilitation, and address contraception.

4 Women-only gatherings intended to promote the sharing of stories and experiences (Longman, 2020).
